# Supplementary material for: Entropy-based dynamic ensemble classication algorithm for imbalanced data stream with concept drift
Source: PLoS One. 2024 Dec 13;19(12):e0311133. doi: 10.1371/journal.pone.0311133 (PMC11643253; doi:10.1371/journal.pone.0311133)

| **数据集** | **REA** | **DWMIL** | **ACDWM** | **MWMOTEB** | **EDAC** | **LPN** |
| --- | --- | --- | --- | --- | --- | --- |
| Gaussian | 0.774085 | 0.855354 | 0.800995 | 0.781268 | 0.866759 | 0.801955 |
| SEA | 0.978214 | 0.976702 | 0.922945 | 0.970823 | 0.981001 | 0.986893 |
| Hyper Plane | 0.638047 | 0.680233 | 0.564992 | 0.549085 | 0.698078 | 0.716552 |
| Checkerboard | 0.839197 | 0.88565 | 0.608972 | 0.872552 | 0.892614 | 0.846563 |
| Electricity | 0.7732 | 0.830261 | 0.618504 | 0.7849 | 0.83033 | 0.839477 |

AUC VALUE

| **数据集** | **REA** | **DWMIL** | **ACDWM** | **MWMOTEB** | **EDAC** | **LPN** |
| --- | --- | --- | --- | --- | --- | --- |
| Moving | 0.219296 | 0.760037 | 0.782664 | 0.543042 | 0.799731 | 0.641783 |
| SEA | 0.892776 | 0.919845 | 0.904575 | 0.921953 | 0.920778 | 0.894144 |
| Hyper Plane | 0.336139 | 0.589351 | 0.549763 | 0.393556 | 0.616357 | 0.492162 |
| Checkerboard | 0.757144 | 0.810838 | 0.596464 | 0.789005 | 0.822574 | 0.751414 |
| Electricity | 0.620795 | 0.706872 | 0.586639 | 0.524369 | 0.717953 | 0.656141 |

GM VALUE

| **数据集** | **REA** | **DWMIL** | **ACDWM** | **MWMOTEB** | **EDAC** | **LPN** |
| --- | --- | --- | --- | --- | --- | --- |
| Moving | 0.1108 | 0.6496 | 0.6374 | 0.3328 | 0.66120 | 0.454000 |
| SEA | 0.8676 | 0.9356 | 0.8964 | 0.908 | 0.9556 | 0.895200 |
| Hyper Plane | 0.147556 | 0.478225 | 0.560755 | 0.211553 | 0.628338 | 0.300766 |
| Checkerboard | 0.709449 | 0.770944 | 0.673529 | 0.676186 | 0.815883 | 0.635258 |
| Electricity | 0.557358 | 0.678736 | 0.65061 | 0.357857 | 0.72899 | 0.593352 |

RECALL VALUE

| **数据集** | **REA** | **DWMIL** | **ACDWM** | **MWMOTEB** | **EDAC** | **LPN** |
| --- | --- | --- | --- | --- | --- | --- |
| Moving | 11.9 | 10.6 | 133.63 | 25.019 | 8.001 | 10.429000 |
| SEA | 45.6 | 32.8 | 224.156 | 59.521 | 24.277 | 32.833000 |
| Hyper Plane | 14.5 | 10.9 | 383.31 | 43.696 | 10.40 | 11.739000 |
| Checkerboard | 282.7 | 54.6 | 272.334 | 176.906 | 50.824 | 145.640000 |
| Electricity | 4.8 | 12.8 | 95.665 | 17.243 | 7.953 | 10.668000 |

TIME


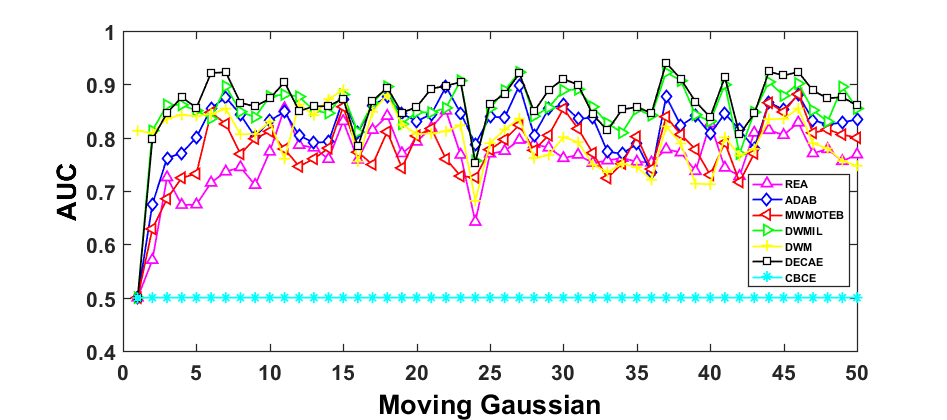


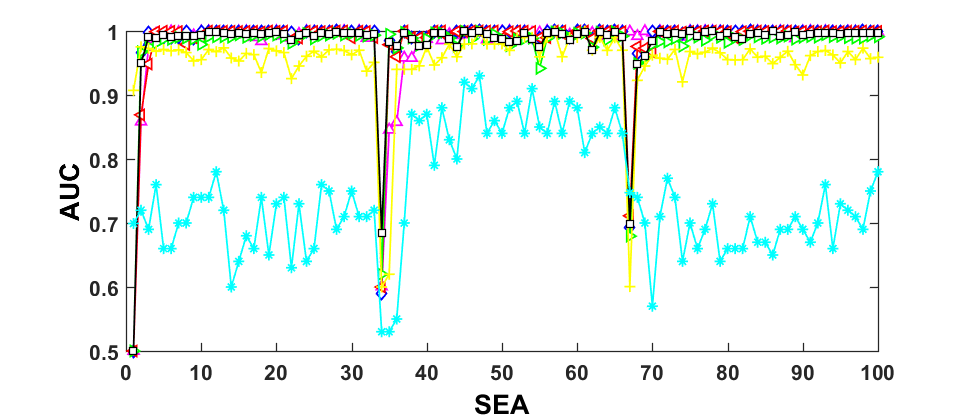


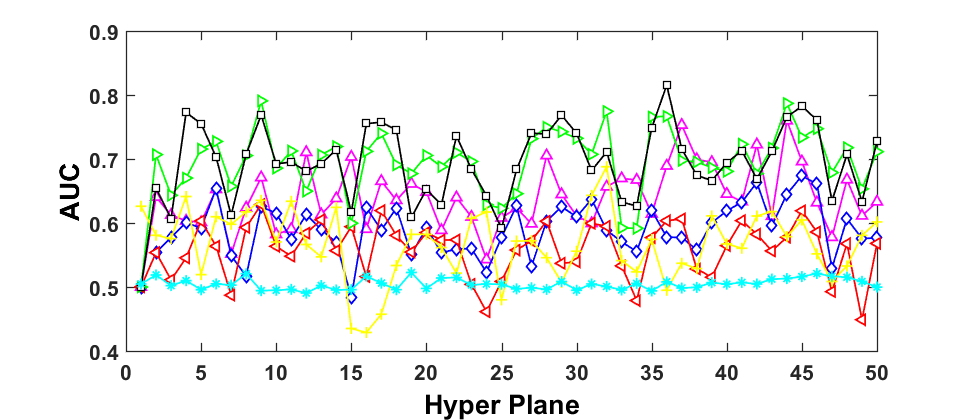


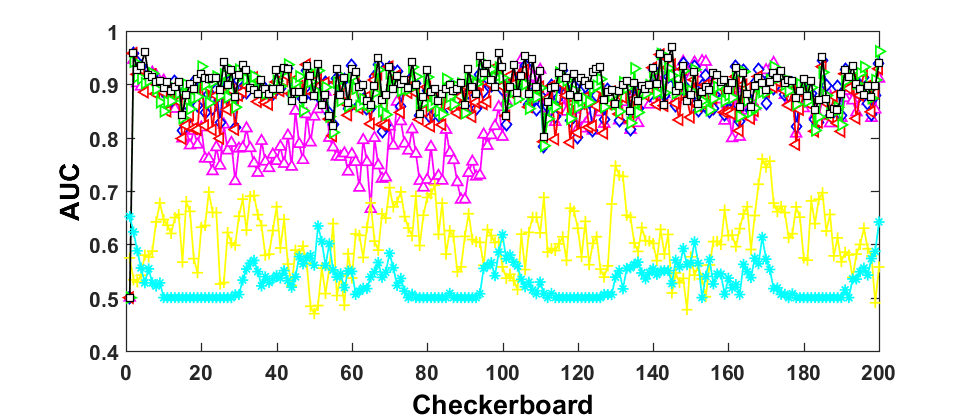


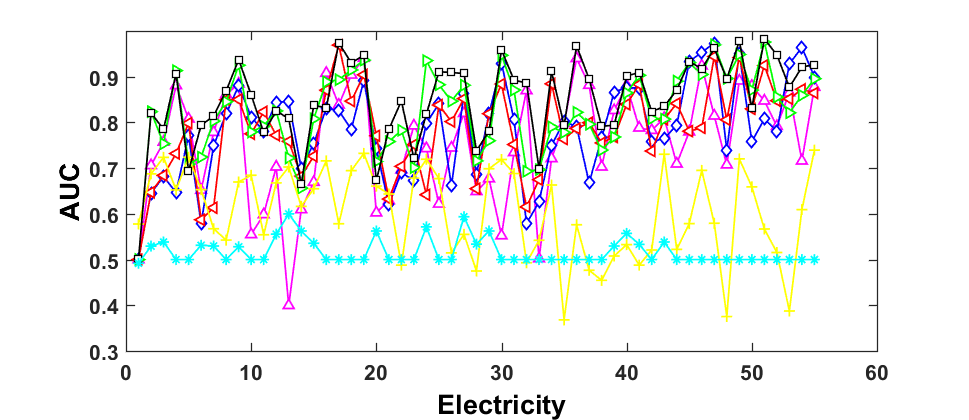


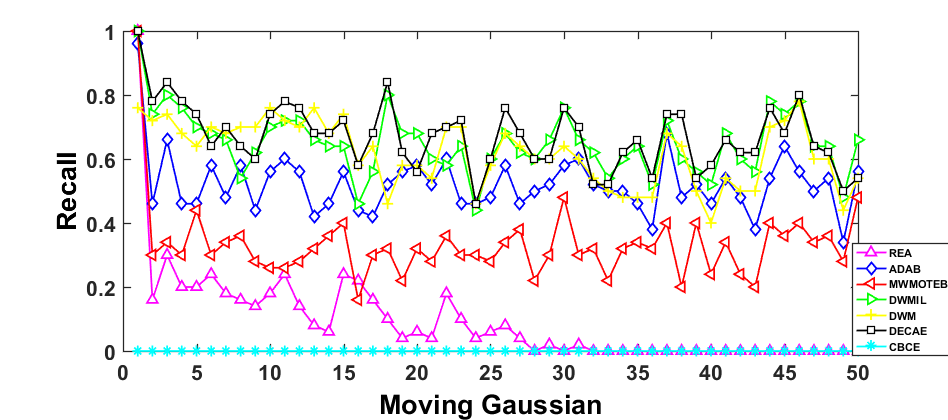


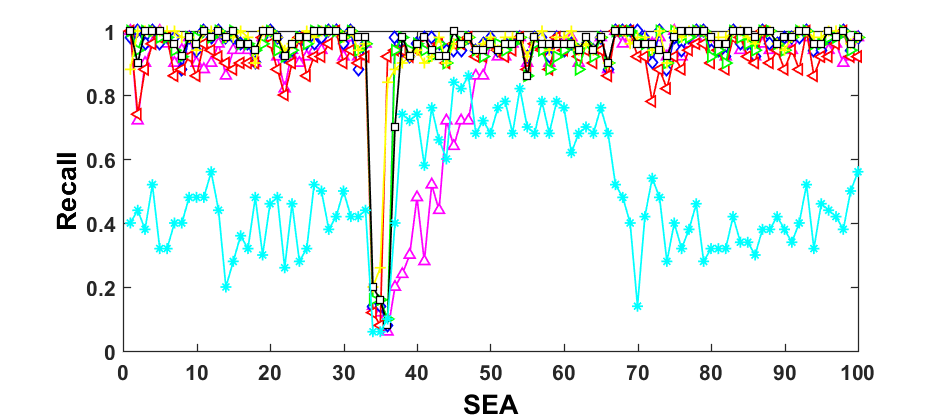


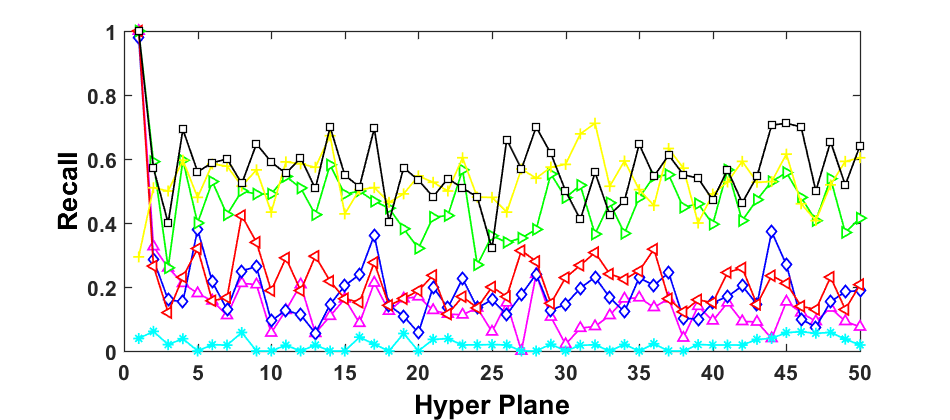


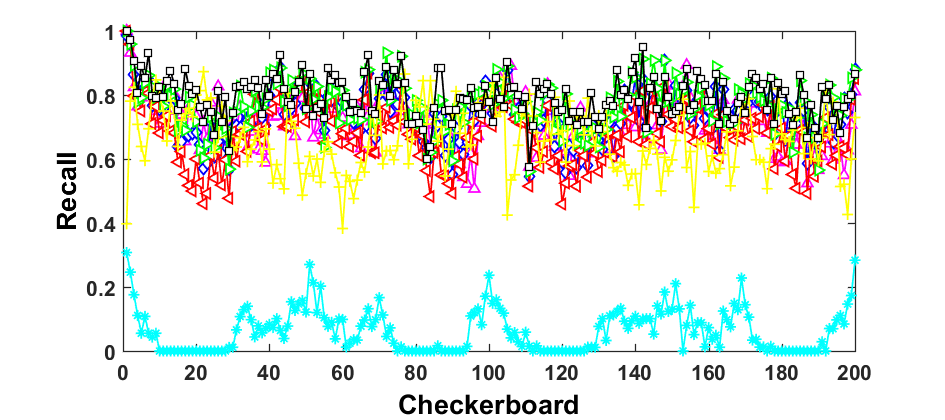


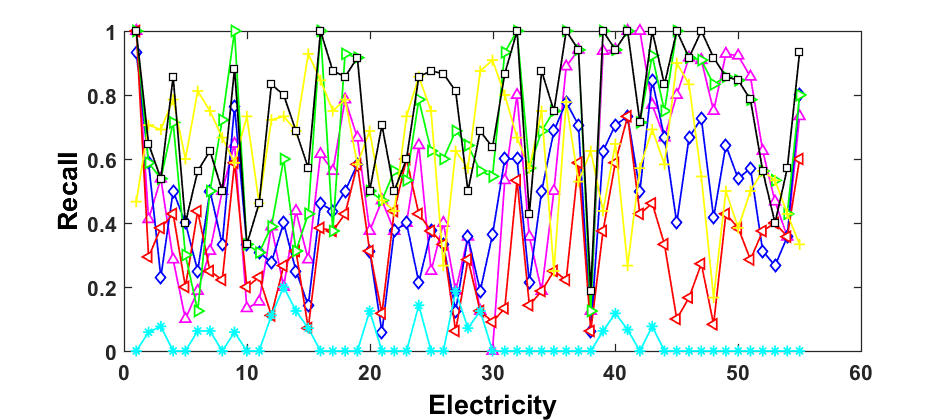

Supplement: S1 File — (DOCX) [file pone.0311133.s006.docx]
