# Supplementary figures and images for: Entropy-based dynamic ensemble classication algorithm for imbalanced data stream with concept drift
Source: PLoS One. 2024 Dec 13;19(12):e0311133. doi: 10.1371/journal.pone.0311133 (PMC11643253; doi:10.1371/journal.pone.0311133)

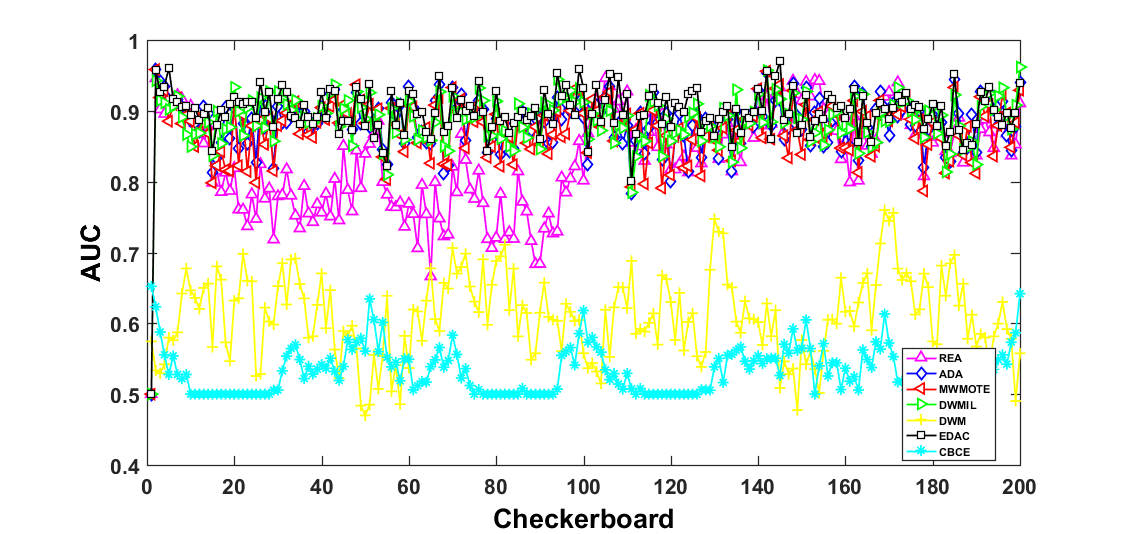

Supplement: S1 Data — (ZIP) [file pone.0311133.s007.zip › Experimental Results Chart/checkauc.png]

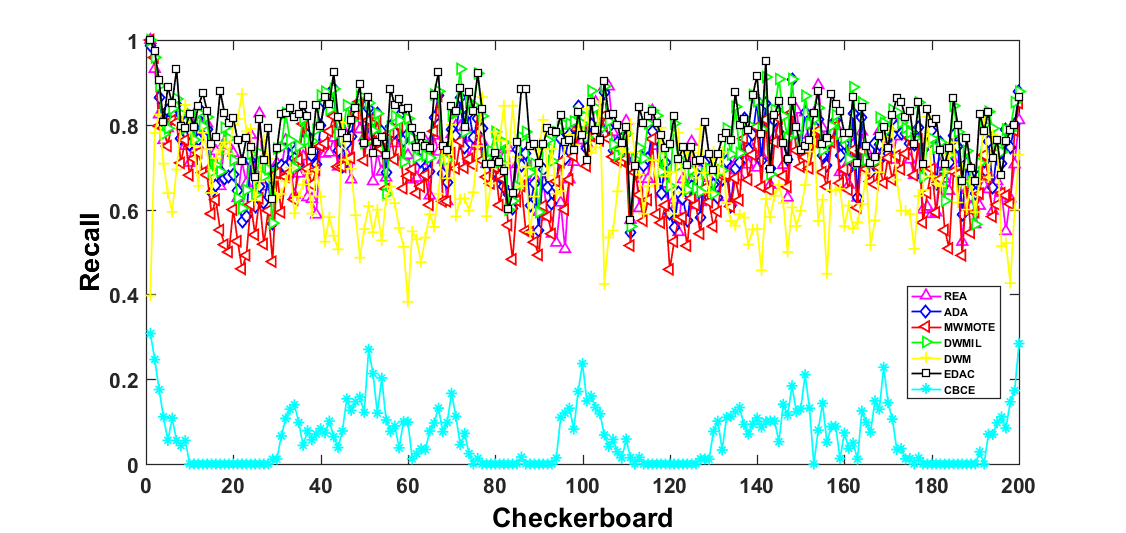

Supplement: S1 Data — (ZIP) [file pone.0311133.s007.zip › Experimental Results Chart/checrecall.png]

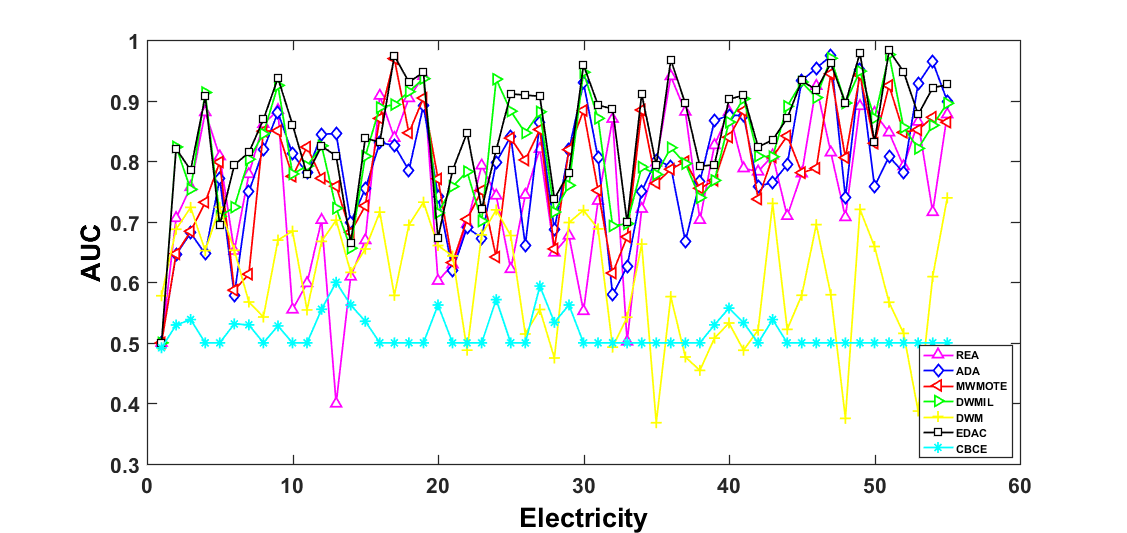

Supplement: S1 Data — (ZIP) [file pone.0311133.s007.zip › Experimental Results Chart/eleauc.png]

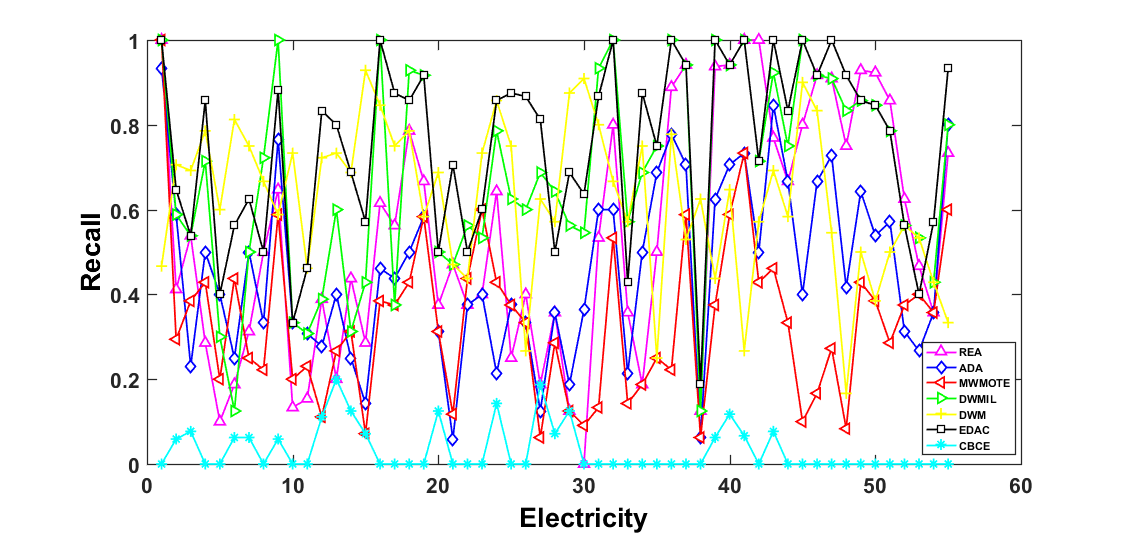

Supplement: S1 Data — (ZIP) [file pone.0311133.s007.zip › Experimental Results Chart/elerecall.png]

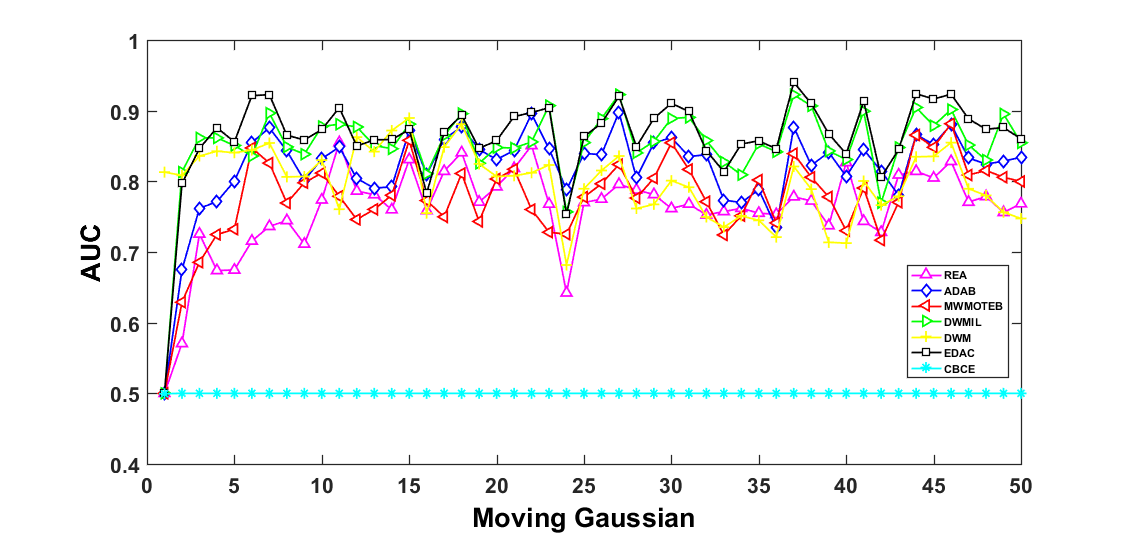

Supplement: S1 Data — (ZIP) [file pone.0311133.s007.zip › Experimental Results Chart/guaauc.png]

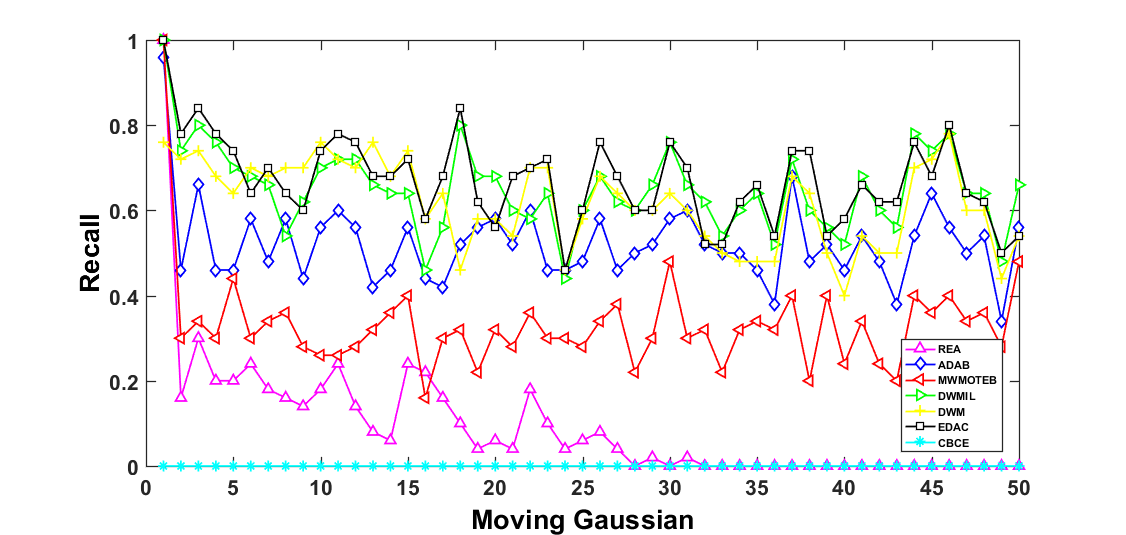

Supplement: S1 Data — (ZIP) [file pone.0311133.s007.zip › Experimental Results Chart/guarecall.png]

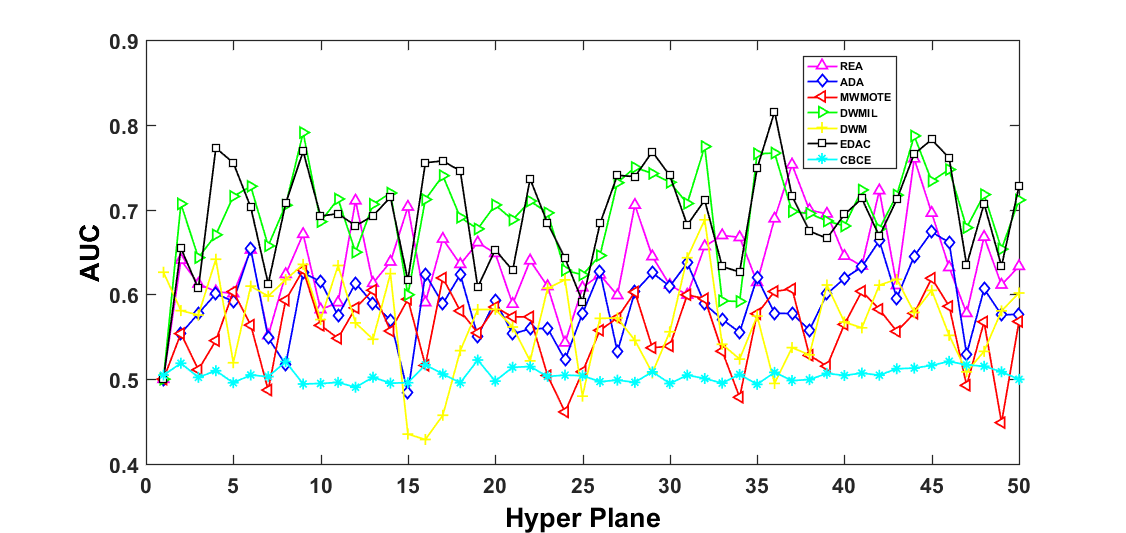

Supplement: S1 Data — (ZIP) [file pone.0311133.s007.zip › Experimental Results Chart/hypauc.png]

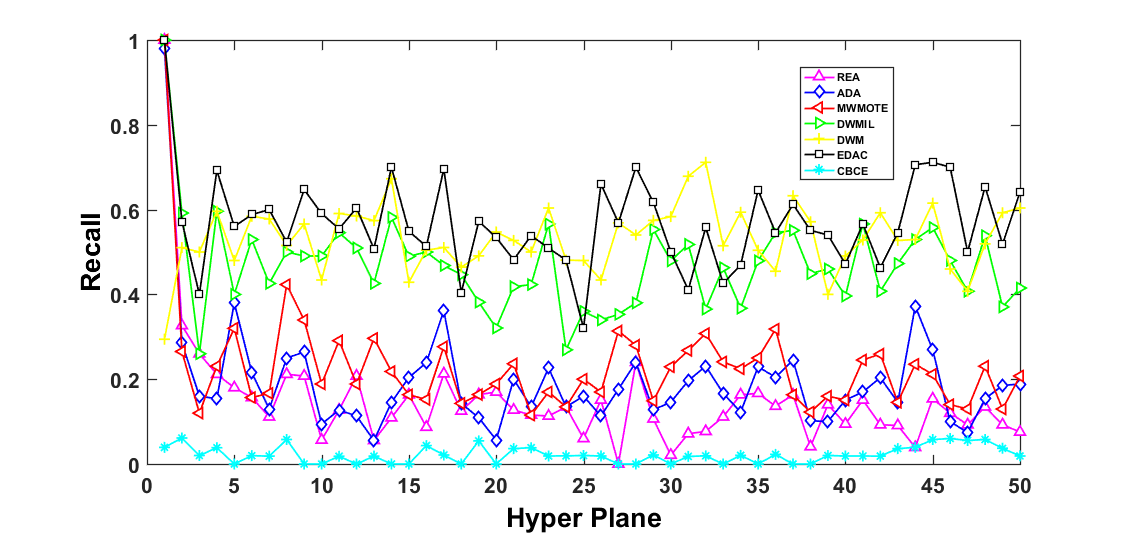

Supplement: S1 Data — (ZIP) [file pone.0311133.s007.zip › Experimental Results Chart/hyprecall.png]

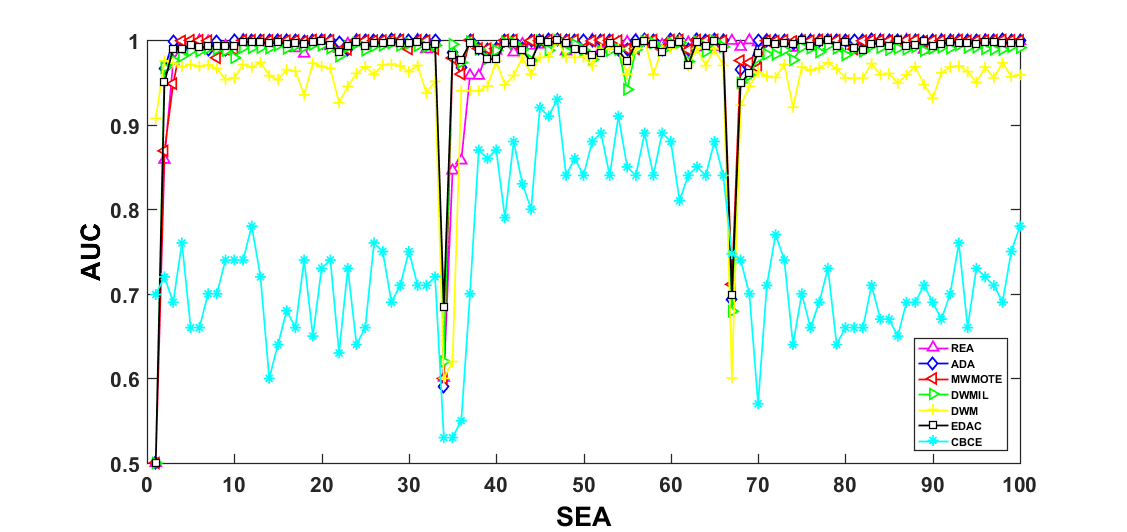

Supplement: S1 Data — (ZIP) [file pone.0311133.s007.zip › Experimental Results Chart/seaauc.png]

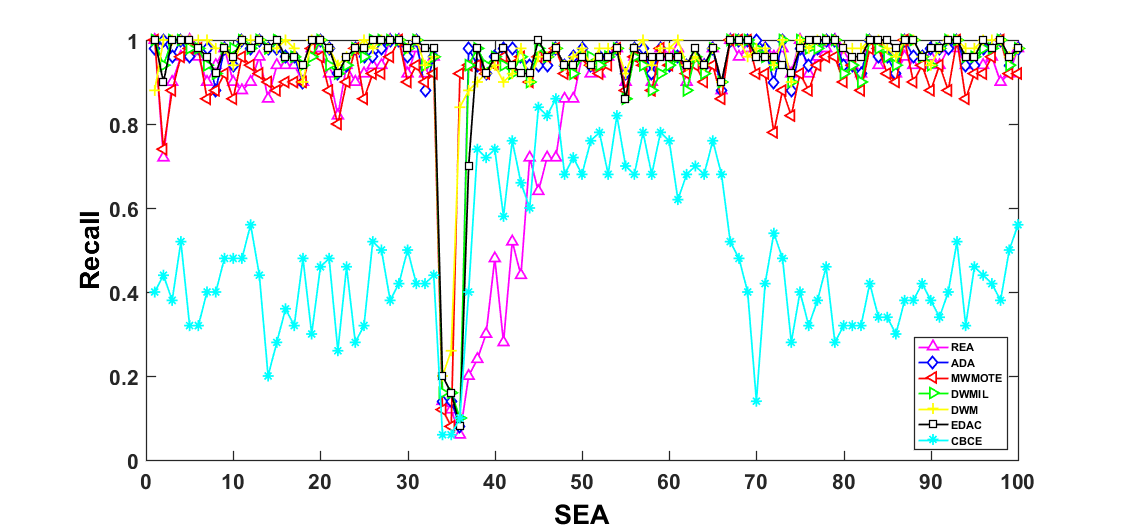

Supplement: S1 Data — (ZIP) [file pone.0311133.s007.zip › Experimental Results Chart/searecall.png]
