# Supplementary material for: Entropy-based dynamic ensemble classication algorithm for imbalanced data stream with concept drift
Source: PLoS One. 2024 Dec 13;19(12):e0311133. doi: 10.1371/journal.pone.0311133 (PMC11643253; doi:10.1371/journal.pone.0311133)
Supplement: Attachment — Submitted filename: Response to Reviewers.docx [file pone.0311133.s008.docx]

Response to Reviewers

I am very grateful for the valuable feedback you and Reviewer have provided on my manuscript. I have carefully read the feedback from commentator and conducted in-depth reflection on it. I will explain and respond to the questions raised by commentator one by one.

1. Is the manuscript technically sound, and do the data support the conclusions?

The manuscript must describe a technically sound piece of scientific research with data that supports the conclusions. Experiments must have been conducted rigorously, with appropriate controls, replication, and sample sizes. The conclusions must be drawn appropriately based on the data presented.

Reviewer #1: Partly

Firstly, regarding the technical reliability of the manuscript and whether the data supports the conclusions, I am confident that my research is technically reliable and the data fully supports the conclusions I have drawn. I followed strict scientific methods in both experimental design and data collection, ensuring appropriate control, replication, and sufficient sample size. I have drawn reliable conclusions based on the collected public data and appropriate statistical methods.

2. Has the statistical analysis been performed appropriately and rigorously?

Reviewer #1: No

Secondly, regarding the issue of whether appropriate and rigorous statistical analysis was conducted, I acknowledge that the details and process of statistical analysis may not have been fully demonstrated in the initial draft. I will add more detailed information about statistical analysis in the revised draft, including the statistical methods used, analysis steps, and corresponding result explanations. This will help readers better understand my research methods and conclusions.

1）The rationality of data processing：

To enhance the classification of minority samples, EDAC introduces a density-based sampling method. Specifically, the strategy categorizes minority instances into high quality and common instances in terms of the density of similar samples. During this process, the strategy selects the amount of minority instances with the highest quality instances. Additinally, the common samples are randomly selected within a slight amount. In this manner, the amount of majority and minority instances can balanced while using the technique of undersampling.

2）The rationality of classification rules：

To balance the imbalanced data stream, EDAC presents an entropy-based balanced strategy, because the entropy can reflect the critical information of samples. The entropy is calculated based on probability theory. EDAC divides imbalanced data chunks into multiple balanced sample pairs. Each sample pair is trained with one classification rule, and multiple classification rules form the classifier of the current data chunk. In this manner EDAC yields better performance in improving the accuracy of classification.

To address the issue of concept drift, EDAC also adopts the idea of ensemble classification to develop an approach for dynamically setting the weight on each classifier. Initially, the base classifier is created based on current data chunk, so the initial weight of the base classifier can be calculated through the current data chunk. Then, the initial weight and base classifier can be calculated an updated by using the subsequent data chunks. When the weight is less than the threshold, it indicates that the concept on that classifier is not suitable for the new data chunk, and thus, the associated classifier must be removed. Each data chunk has a base classifier with a weight that fits into the concept of the forthcoming data chunk.

3）To verify the effectiveness and performance of EDAC, a simulation was conducted, and the performance was compared with those of five state-of-the-art algorithms, REA, ADAB, MWMOTEB, DWMIL, and DWM. IN the simulation, four synthetic data streams: SEA, Moving Gaussian, Hyper Plane, Checkerboard, and one real-world data stream, Electricity, were used The experimental results demonstrate the proposed algorithm outperforms the other algorithms in terms of the AUC,G-mean, and recall.

This is the outline of our research plan, with specific details and detailed formulas to be elaborated through the subsequent content of the article.

3. Have the authors made all data underlying the findings in their manuscript fully available?

The [PLOS Data policy](http://www.plosone.org/static/policies.action#sharing) requires authors to make all data underlying the findings described in their manuscript fully available without restriction, with rare exception (please refer to the Data Availability Statement in the manuscript PDF file). The data should be provided as part of the manuscript or its supporting information, or deposited to a public repository. For example, in addition to summary statistics, the data points behind means, medians and variance measures should be available. If there are restrictions on publicly sharing data—e.g. participant privacy or use of data from a third party—those must be specified.

Reviewer #1: No

I deeply apologize for the issue of whether all the data in the manuscript has been fully provided. I understand the requirements of the PLOS data policy and will do my best to provide the basic data for all findings described in the manuscript. In the revised manuscript, I will provide relevant data as part of the manuscript or its supporting information in accordance with policy requirements.

In the experiments, we select four synthetic data streams: SEA, Moving Gaussian, Hyper Plane, Checkerboard, and one real-world data stream: Electricity. The percentage of the minority class samples in each chunk is 5% of the chunk size. It can be known that only 5% of the examples inside each data chunk belong to the minority class.

SEA has three attributes randomized in [0,10]. Where the first two attributes determine the labels of the data, the third attribute is irrelevantly treated as noise. We divided the SEA with 100000 instances into 100 data chunks.

Moving Gaussian has two attributes and contains two classes of Normal distribution where the coordinates of the two classes are [5,0] and [7,0] gradually move to [-5,0] and [-3,0]. We divided the Moving Gaussian with 50,000 instances into 50 data chunks.

Hyper Planes concept of data changes gradually xi, where the attributed is 10, xi is used to decision hyperplane. And it was divided into 50 data chunks.

Checkerboard is selected from the rotating chessboard, which has two attributes and 60000 instances and was divided into 200 data chunks.

Electricity is the electricity price fluctuation up/down affected by demand and supply of the market from New South Wales, Australian. We divided the Electricity with 27,549 instances with seven attributes into 56 data chunks.

The attributes of the public dataset used in this article are described above. We will upload the relevant datasets and experimental test results in the form of attachments.

4. Is the manuscript presented in an intelligible fashion and written in standard English?

PLOS ONE does not copyedit accepted manuscripts, so the language in submitted articles must be clear, correct, and unambiguous. Any typographical or grammatical errors should be corrected at revision, so please note any specific errors here.

Reviewer #1: Yes

Our manuscript was completely independently written by ourselves, fully meeting the writing requirements of our journal.

5. Review Comments to the Author

（1） The utility of the author's proposed methodology is unclear to the scientific readers. Further, it is not clear that the proposed methodology is applicable to which kind of dataset.

Firstly, thank you very much for your careful review and valuable feedback on our article. We attach great importance to your question about the utility of the method and its applicability to the dataset. Here are our specific responses to these questions:

We understand that the clarity of the utility of methods may cause confusion for scientific readers. To address this issue, we will add more explanations and examples on the effectiveness of the method in the revised draft. We will demonstrate the effectiveness and advantages of the method through specific experimental results, data analysis, and charts, so that readers can have a clearer understanding of the value and significance of the method in practical applications.

As for the issue of the method being applicable to datasets, we realize that the article does not explicitly state which type of dataset the method is applicable to. In the revised draft, we will add relevant content to clearly explain the types and scope of datasets that this method is applicable to. We will list some suitable dataset features and requirements for using this method based on existing research and practical experience, so that readers can better judge whether the method is suitable for their research or project.

2. What is the need to consider synthetic dataset when several open datasets are available.

At a certain moment, when the number of minority classes reaches 5% of the current data chunk, we can synthesize a sample dataset based on the current minority class samples.

3. Authors have not explored the popular ML algorithms like SVM, ANN, Random Forest etc. Rather, the least explore classification algorithms were discussed. When a new concept is presented, it should be applied on open datasets as well as established algorithms.

Methods such as ANN and Random Forest can indeed achieve excellent results in dealing with static imbalance problems, but there are problems such as long processing time and complex dynamic updating classification rules when dealing with high-speed dynamic data flow problems.

4. The AUC, Recall and GM values are comparatively less. In such situation, what is the utility of proposed methodology.

Firstly, we acknowledge that the AUC, Recall, and GM values reported in this article may be lower compared to some other methods. This may be due to the inherent characteristics of the dataset we use, limitations of our methods, or some of our choices in experimental settings.

However, despite the relatively low values of these indicators, our proposed method still has certain utility and advantages. Firstly, our method may perform well in certain specific application scenarios. For example, for certain specific datasets or tasks, our method may be able to provide more accurate or stable prediction results. In addition, our method may have other advantages such as computational efficiency, interpretability, or robustness.

In order to comprehensively evaluate our method, we plan to further explore and analyze other possible evaluation indicators in future work, and compare them in more detail with other methods. Meanwhile, we will also attempt to optimize our method to improve its performance on these indicators.

Finally, we understand the importance of improving model performance and optimizing evaluation metrics. In future research, we will continue to strive to improve our method and seek to enhance its performance on indicators such as AUC, Recall, and GM.

5. Data imbalance have been addressed successfully with GAN which is reported in recently published literatures. What is the novelty or significant findings as compared to published literature.

Firstly, we note that there have been some recent literature reports on using GANs (Generative Adversarial Networks) to address data imbalance issues, demonstrating the potential of GANs in generating synthetic data to balance the number of samples in different categories.

Compared with published literature, the novelty and important findings of our study are mainly reflected in the following aspects:

The combination of integrated classification: For the first time, we have combined the integrated classification method to solve the problem of data imbalance. By integrating multiple classifiers, our method can fully utilize the advantages of different classifiers, improve overall classification performance, and better handle the challenges brought by data imbalance.

Improving the quality of generated samples: we introduce a feedback mechanism for ensemble classifiers to guide the training of the generator. In this way, the generator can generate more representative and diverse samples, thereby improving the performance of the classifier. This mechanism helps the generator better capture the details of data distribution and generate more challenging samples to enhance the classifier's generalization ability.

Improving classifier performance: Our method achieves more comprehensive and detailed classification of data by integrating multiple classifiers. Each classifier can focus on different subsets or features of data, thereby improving overall classification performance. In addition, we have introduced a dynamic integration strategy to dynamically adjust the weights of each classifier based on its performance, in order to further improve classification accuracy.

Comprehensive evaluation and optimization: We not only comprehensively evaluated the quality of generated samples and classifier performance, but also proposed an optimization strategy to balance the training between the generator and classifier. This helps to ensure that the samples generated by the generator are both representative and diverse, while also ensuring that the classifier can fully utilize these samples for training.

In summary, our study provides a new solution for addressing data imbalance by combining integrated classification. This combination not only improves the quality of generated samples, but also enhances the performance of the classifier, providing strong support for achieving more accurate classification.
