# Supplementary material for: Entropy-based dynamic ensemble classication algorithm for imbalanced data stream with concept drift
Source: PLoS One. 2024 Dec 13;19(12):e0311133. doi: 10.1371/journal.pone.0311133 (PMC11643253; doi:10.1371/journal.pone.0311133)
Supplement: Attachment — Submitted filename: Response to Reviewers.docx [file pone.0311133.s009.docx]

Dear reviewer,

Response to Reviewers

I am very grateful for the valuable feedback you and Reviewer have provided on my manuscript. I have carefully read the feedback from commentator and conducted in-depth reflection on it. I will explain and respond to the questions raised by commentator one by one.

4. Have the authors made all data underlying the findings in their manuscript fully available?

The [PLOS Data policy](http://www.plosone.org/static/policies.action#sharing) requires authors to make all data underlying the findings described in their manuscript fully available without restriction, with rare exception (please refer to the Data Availability Statement in the manuscript PDF file). The data should be provided as part of the manuscript or its supporting information, or deposited to a public repository. For example, in addition to summary statistics, the data points behind means, medians and variance measures should be available. If there are restrictions on publicly sharing data—e.g. participant privacy or use of data from a third party—those must be specified.

The dataset mentioned in the document is in Attachment 《dataset》, the data points behind the mean, median, and variance measures in the experiment are shown in Appendix 《Experimental Results Chart》. And we provided explanations for obtaining the dataset in the manuscript

I deeply apologize for the issue of whether all the data in the manuscript has been fully provided. I understand the requirements of the PLOS data policy and will do my best to provide the basic data for all findings described in the manuscript. In the revised manuscript, I will provide relevant data as part of the manuscript or its supporting information in accordance with policy requirements.

In the experiments, we select four synthetic data streams: SEA, Moving Gaussian, Hyper Plane, Checkerboard, and one real-world data stream: Electricity. The percentage of the minority class samples in each chunk is 5% of the chunk size. It can be known that only 5% of the examples inside each data chunk belong to the minority class.

SEA has three attributes randomized in [0,10]. Where the first two attributes determine the labels of the data, the third attribute is irrelevantly treated as noise. We divided the SEA with 100000 instances into 100 data chunks.

Moving Gaussian has two attributes and contains two classes of Normal distribution where the coordinates of the two classes are [5,0] and [7,0] gradually move to [-5,0] and [-3,0]. We divided the Moving Gaussian with 50,000 instances into 50 data chunks.

Hyper Planes concept of data changes gradually xi, where the attributed is 10, xi is used to decision hyperplane. And it was divided into 50 data chunks.

Checkerboard is selected from the rotating chessboard, which has two attributes and 60000 instances and was divided into 200 data chunks.

Electricity is the electricity price fluctuation up/down affected by demand and supply of the market from New South Wales, Australian. We divided the Electricity with 27,549 instances with seven attributes into 56 data chunks.

The attributes of the public dataset used in this article are described above. We will upload the relevant datasets and experimental test results in the form of attachments.

5. Is the manuscript presented in an intelligible fashion and written in standard English?

PLOS ONE does not copyedit accepted manuscripts, so the language in submitted articles must be clear, correct, and unambiguous. Any typographical or grammatical errors should be corrected at revision, so please note any specific errors here.

Thank you for raising the question about the manuscript's presentation and language. We have taken great care to ensure that our manuscript is presented in an intelligible fashion and written in standard English.

Our manuscript was completely independently written by ourselves, fully meeting the writing requirements of our journal. Our manuscript has been polished by the Editage and revised based on the feedback provided.

6. Review Comments to the Author

Reviewer #3:

1. Entropy-based dynamic ensemble classification algorithm (EDAC) are not clearly articulated. You need to provide more compelling evidence and explanations to demonstrate the advantages of your approach over existing methods and clarify the specific types of datasets it is suited for.

Firstly, thank you very much for your careful review and valuable feedback on our article. We attach great importance to your question about the utility of the method and its applicability to the dataset. Here are our specific responses to these questions:

We understand that the clarity of the utility of methods may cause confusion for scientific readers. To address this issue, we will add more explanations and examples on the effectiveness of the method in the revised draft. We will demonstrate the effectiveness and advantages of the method through specific experimental results, data analysis, and charts, so that readers can have a clearer understanding of the value and significance of the method in practical applications.

As for the issue of the method being applicable to datasets, we realize that the article does not explicitly state which type of dataset the method is applicable to. In the revised draft, we will add relevant content to clearly explain the types and scope of datasets that this method is applicable to. We will list some suitable dataset features and requirements for using this method based on existing research and practical experience, so that readers can better judge whether the method is suitable for their research or project.

2. In your related work section and discussion, you should provide a more thorough comparison of EDAC with these GAN-based methods, focusing on the novelty and specific advantages of your approach.

Firstly, we note that there have been some recent literature reports on using GANs (Generative Adversarial Networks) to address data imbalance issues, demonstrating the potential of GANs in generating synthetic data to balance the number of samples in different categories. But GANs mainly solves the problem of static imbalanced data classification. As it is a neural network algorithm, it takes a long time to process high-speed data streams and is not suitable for solving dynamic data stream classification problems.

Compared with published literature, the novelty and important findings of our study are mainly reflected in the following aspects:

The combination of integrated classification: For the first time, we have combined the integrated classification method to solve the problem of data imbalance. By integrating multiple classifiers, our method can fully utilize the advantages of different classifiers, improve overall classification performance, and better handle the challenges brought by data imbalance.

Improving the quality of generated samples: we introduce a feedback mechanism for ensemble classifiers to guide the training of the generator. In this way, the generator can generate more representative and diverse samples, thereby improving the performance of the classifier. This mechanism helps the generator better capture the details of data distribution and generate more challenging samples to enhance the classifier's generalization ability.

Improving classifier performance: Our method achieves more comprehensive and detailed classification of data by integrating multiple classifiers. Each classifier can focus on different subsets or features of data, thereby improving overall classification performance. In addition, we have introduced a dynamic integration strategy to dynamically adjust the weights of each classifier based on its performance, in order to further improve classification accuracy.

Comprehensive evaluation and optimization: We not only comprehensively evaluated the quality of generated samples and classifier performance, but also proposed an optimization strategy to balance the training between the generator and classifier. This helps to ensure that the samples generated by the generator are both representative and diverse, while also ensuring that the classifier can fully utilize these samples for training.

In summary, our study provides a new solution for addressing data imbalance by combining integrated classification. This combination not only improves the quality of generated samples, but also enhances the performance of the classifier, providing strong support for achieving more accurate classification.

3. You need to provide a more comprehensive analysis of your method's performance, discuss the implications of these results, and outline strategies for improvement. Optionally, Consider exploring alternative evaluation metrics that may better highlight the strengths of your approach.

Thank you very much for carefully reviewing our paper and providing valuable feedback. We fully agree with your viewpoint on the need for a more comprehensive analysis of method performance, discussion of the impact of results, and suggestions for improvement. We have added an explanation of the meaning of the results and outlined improvement strategies in 2.3 Comparison and Analysis：These three indicators evaluate the performance of classification models from different perspectives. AUC provides information about the overall discriminative ability of the classifier, G-Means helps balance accuracy and recall, especially in cases of imbalanced categories, while Recall focuses particularly on the completeness of positive example recognition. Our algorithm EDAC may have lower GM values than other algorithms in certain specific situations. Overall, our algorithm EDAC has achieved excellent performance in all three indicators.In the later stage, we will increase the number of sample points and sacrifice a small amount of time to increase the accuracy of the algorithm.

Thank you again for your valuable feedback and attention. We look forward to receiving your further guidance and support.

Best regards,

Jiaming Gong
