# Supplementary material for: Entropy-based dynamic ensemble classication algorithm for imbalanced data stream with concept drift
Source: PLoS One. 2024 Dec 13;19(12):e0311133. doi: 10.1371/journal.pone.0311133 (PMC11643253; doi:10.1371/journal.pone.0311133)
Supplement: Attachment — Submitted filename: Response to Reviewers.docx [file pone.0311133.s010.docx]

Dear reviewer,

Response to Reviewers

I am very grateful for the valuable feedback you and Reviewer have provided on my manuscript. I have carefully read the feedback from commentator and conducted in-depth reflection on it. I will explain and respond to the questions raised by commentator one by one.

3. Has the statistical analysis been performed appropriately and rigorously?

Descriptive Statistical Analysis and Performance Metrics:

Indeed, we have employed descriptive statistical analysis methods, specifically by calculating the Area Under the Curve (AUC), Recall, and G-Means, to comprehensively evaluate the performance of our proposed model, EDAC. These metrics were chosen as they provide a multi-faceted view of the model's classification capabilities:

AUC quantifies the model's overall discriminative power, giving a measure of how well the model can distinguish between classes across different decision thresholds.

Recall emphasizes the ability of the model to identify all positive instances, crucial in applications where false negatives are costly.

G-Means helps assess the trade-off between precision and recall, addressing the challenge of imbalanced datasets by striving for a balance between these two aspects of performance.

Rigorousness and Limitations:

While these metrics offer a robust foundation for evaluating our model, we acknowledge that no single metric can fully encapsulate all aspects of model performance. Therefore, we have taken a multi-metric approach to ensure a comprehensive assessment.

6. Review Comments to the Author

(1)The suggested algorithms need to be updated in detail.

We acknowledge that a more detailed description of the proposed algorithms is essential for readers to fully understand and replicate our work. In the revised manuscript, we have expanded Section 1 The proposed algorithm: EDAC , to provide a step-by-step explanation of the Entropy-based Dynamic Ensemble Classification Algorithm. This includes a detailed algorithm flowchart, pseudo-code, and explanations of each component's functionality and purpose. We have also included additional figures and examples to illustrate the algorithm's working principles and decision-making processes.

(2) Theoretical analysis is neexed. The theoretical complexity analysis and comparision with the state of the srt approach is needed.

We fully agree that a thorough theoretical analysis and comparison with the state-of-the-art approaches are crucial to demonstrate the merits and positioning of our work. In response to your suggestion, we have strengthened Section 2.2 "Theoretical Analysis and Algorithm Comparison" by including a detailed examination of the time and space complexity of our algorithm, as well as a comprehensive comparison with the most recent and relevant algorithms in the field.

Conducted a Rigorous Complexity Analysis: We have carefully analyzed the time and space complexity of our algorithm, taking into account the key factors that influence its performance, such as the input data size, problem dimensionality, and any inherent parallelism or optimization strategies employed. This analysis provides a clear picture of how our algorithm scales with respect to these parameters, enabling readers to assess its suitability for various applications and datasets.

(3)Recent related references published on 2024, 2023, 2022 need to be added and analyzed.

We appreciate your suggestion to include recent references from 2024, 2023, and 2022. In the revised manuscript, we have thoroughly reviewed the latest literature in the field and added relevant references to Introduction, where we discuss related work. We have analyzed these recent publications, discussing how they relate to and differ from our proposed approach. This update ensures that our work is situated within the most current research landscape.

(4)There are still inconsistent sentences. Tight proofreading is needed.

We apologize for any inconsistencies or unclear sentences in the original manuscript. We have undertaken a rigorous proofreading process, carefully reviewing each sentence for clarity, coherence, and grammatical correctness. We have corrected any errors identified and ensured that the revised manuscript is consistent in its terminology, notation, and explanations. We believe that these changes have significantly improved the readability and accessibility of our work.

In conclusion, we are grateful for your insightful comments and suggestions, which have helped us improve the quality and impact of our manuscript. We hope that the revisions we have made address your concerns and that the revised version meets your expectations. We look forward to your further feedback and the opportunity to clarify any remaining questions you may have.

Thank you again for your valuable feedback and attention. We look forward to receiving your further guidance and support.

Best regards,

Jiaming Gong
